# Understanding the role of cannabis in patients with suicidal ideation presenting to the emergency department: systematic chart review

**DOI:** 10.1192/bjo.2025.10776

**Published:** 2025-09-09

**Authors:** Maria Simmons, Candice E. Crocker, Derek Fisher, Sherry H. Stewart, Jason G. Emsley, Sabina Abidi, Alix Carter, Cynthia Calkin, Igor Yakovenko, Kirk Magee, Philip G. Tibbo

**Affiliations:** Department of Psychiatry, Dalhousie University, Halifax, Nova Scotia, Canada; Department of Diagnostic Radiology, Dalhousie University, Halifax, Nova Scotia, Canada; Department of Psychology, Mount St. Vincent University, Halifax, Nova Scotia, Canada; Department of Psychology and Neuroscience, Dalhousie University, Halifax, Nova Scotia, Canada; Department of Emergency Medicine, Dalhousie University, Halifax, Nova Scotia, Canada; IWK Children’s Health Center, Halifax, Nova Scotia, Canada; Department of Emergency Medicine, Nova Scotia Health, Halifax, Nova Scotia, Canada; Department of Psychiatry, Nova Scotia Health, Halifax, Nova Scotia, Canada

**Keywords:** Suicide, cannabis use, emergency, adverse event

## Abstract

**Background:**

The association between cannabis use and suicidality has been established, but details on impacts of legalisation, as well as long-term service use, have had limited attention.

**Aims:**

To examine if changes are present in suicide presentations with access to legal cannabis.

**Method:**

This study employed administrative database and medical record reviews to identify two cohorts of patients presenting with suicidal ideation/attempts and cannabis use to emergency departments, for two periods: 17 October 2018 to 30 April 2019, and 17 October 2020 to 30 April 2021. Demographic and clinical outcome data were obtained, and emergency department healthcare usage for 2 years before and 2 years after index encounter were compared, to further understand emergency department presentations for the same complaint.

**Results:**

Number of emergency department encounters following the index visit and number of emergency department encounters specifically relating to suicidality following the index visit were significantly different between cohorts (*t* = 2.05, *P* = 0.042; *t* = 2.23, *P* = 0.027, respectively), with the immediate post-cannabis legalisation period demonstrating greater numbers of subsequent emergency department visits for suicidality. Additional associations were found between personality disorders and repeat emergency department visits related to cannabis use.

**Conclusions:**

There appears to be stability in the patient profile of those presenting to the emergency department with a complaint relating to suicide while reporting cannabis use from the period directly following legalisation in Canada, to a similar time frame 2 years later despite reported increased use of cannabis in the general population over this period. Despite the rising potency and access to legal cannabis, suicide risk remains stable, although concerning.

Cannabis use disorder (CUD), daily cannabis use and non-daily cannabis use have all been associated with a higher prevalence of suicidal ideation, plans and attempts (i.e. suicidality).^1–5^ However, this association of increased risk of suicidality and cannabis use is not consistently reported.^6^ These contradictions may relate to outcomes for specific groups of cannabis users. Compared with individuals who do not use substances, individuals with a CUD have been found to be 2.01 times more likely to experience suicidal ideation in the past 12 months and are 1.71 times more likely to have had a suicide attempt.^7^ CUD may not be the only marker. Adolescents with non-disordered cannabis use have been found to have 2.08 times higher odds of suicidal ideation than non-users.^8^ This suggests an association between suicidal ideation and both disordered and non-disordered cannabis use, with particular concern in younger individuals. These studies focused on suicidal ideation, but notably, there is a systematic review and meta-analysis reporting that suicidal ideation may be just as likely as suicidal behaviour in predicting later suicide.^9^ Also, in individuals who have suicidal thoughts or practice non-suicidal self-harm, cannabis use is one of the strongest predictors of transition to suicide attempt.^10^ Collectively, these data suggest that any level of cannabis use may be associated with suicidal behaviours, but this has not been well studied.

Post-mortem toxicology reports can be instructive regarding the link between cannabis use and completed suicide, by helping determine the frequency of cannabis involvement in deaths involving suicide. One such study reported up to 30% of individuals who died by suicide had positive toxicology reports for cannabis, with trends showing an increase in positive cannabis toxicology reports since 2004.^5^ This finding has also been supported by research using the US National Poison Data system, which found increasing reports of suspected suicidal ideation or attempts associated with cannabis exposures between 2009 and 2021.^3^ In Canada, where recreational cannabis was legalised in 2018, a study in the province of New Brunswick showed a significant increase in the proportion of cannabis-positive serology reports in suicidal deaths from pre- to post-legalisation (18.1–30.7%^4^). While acknowledging the possible confound of the long half-life of cannabis, and thus the difficulty in assessing cannabis intoxication at time of death, this significant jump in proportions nonetheless remains a cause for concern. A clearer picture would be possible with examining patients who are acutely suicidal or have a suicide attempt. These individuals are often found in emergency department populations.

Recent reviews of the literature with respect to cannabis presentations to emergency departments have emphasised that, for many patients, the first point of contact with healthcare following an acute adverse event related to cannabis use is with the emergency department.^11,12^ A retrospective chart review in Colorado, USA, a state that legalised recreational cannabis use in 2014, found cannabis-related presentations to the emergency department increased over time and were more likely to result in negative outcomes such as admissions to hospital.^13^ The same study also examined cannabis-related presentations to the emergency department specifically related to psychiatric presentations and found that 18.6% were related to a suicide attempt and 2.1% to suicidal ideation, with suicidality making up making up over 20% of all acute psychiatric complaints following cannabis use.^13^

The literature on the reported rates of emergency department presentations with cannabis-related complaints overall in Canada since legalisation has been inconsistent; some research has suggested a rise in all-cause cannabis-related emergency department visits,^14–16^ whereas other studies have not supported this.^17^ These mixed findings may be attributable to methodological variability and differences in emergency department reporting systems.^12,18^ These studies also tend to be cohort studies without examination of follow-up visits for any further healthcare utilisation, and often without any indication of number of return emergency department visits over time. This study approach potentially downplays the impact of emergency department visits related to cannabis on the healthcare system.

Cannabis use increased in Canada during the COVID-19 pandemic.^19^ One study examining the impact of cannabis use on suicidal ideation during the COVID-19 pandemic reported that, after controlling for psychosocial variables and other confounding variables, cannabis consumption was significantly associated with suicidal ideation.^20^ Another study of 9470 cases of substance use-related self-harm in one emergency department, cannabis alone was involved in only 1.7% of self-harm cases, but cannabis in combination with other substance was related to 20% of self-harm cases.^21^ Importantly, because of the potential issues of coding for emergency department administrative databases that may not adequately collect chronic cannabis use or use that is perceived as medical by the patient, a mixed emergency department database and more in-depth chart review methodological approach is warranted to ensure completeness of data. This absence of studies examining long-term outcomes and the pathway to care for these patients (e.g. number of emergency department visits before an index visit for suicidality and cannabis use, and subsequent emergency department visits) makes modelling the impact of cannabis use on healthcare utilisation and knowledge translation regarding cannabis risks challenging. To address this deficiency, we examined emergency department visits for two 6-month time periods to create two cohorts, with the first cohort anchored to the day after legalisation and the second commencing on the same date 2 years later, which were then followed for subsequent service use. We also took a relatively novel in-depth look at the patient mental health diagnoses, if present, and other patient-level characteristics to examine if any of these factors were an identifiable risk factor associated with return emergency department visits.

## Method

### Study population

This study examined a cohort of patients that comprised a subset of a larger study which manually screened emergency department admissions between two 6-month time frames to select encounters where patients had reported recent cannabis use.^22^ This substudy employed administrative database and chart review methodology of individuals presenting to the four main adult emergency departments in Nova Scotia Health Central Zone, Canada (Hants Community Hospital, Halifax Infirmary, Dartmouth General and Cobequid Community Health Centre). These four emergency departments had a catchment area population of approximately 430 000 at the time of this study. This study received clearance from the Nova Scotia Health Research Ethics Board (protocol number 1026418). The requirement for informed consent was waived because of the study’s secondary use of data and reporting of data in aggregate form only.

An administrative database review using ICD-9^23^ codes identified 43 957 unique patient encounters meeting screening criteria of potential cannabis-related presentations (mental or physical health issues). These encounters were from two 6-month time frames starting at the time of legalisation in Canada (17 October 2018) to 30 April 2019, and then 2 years later from 17 October 2020 to 30 April 2021 (comparator time frame). The later April closing date was chosen to encompass presentations related to 20 April (4 20) – a date well known for being associated with cannabis advocacy. The cohorts were to examine the expected increase in cannabis-related presentations seen after legalisation in various jurisdictions including Uruguay and certain states within the USA.^24–26^ The second cohort also overlaps a time period during which COVID-19 public health restrictions were still in place in our jurisdiction, but cannabis use had also increased during this period.^19^ The patient encounters identified by an administrative database search during this period were then examined by manually performing chart reviews to confirm if the patient had consumed cannabis in any form for any reason within 24 h of the presentation or identified themselves as taking cannabis as a medication. This reduced the 43 952 encounters to a cohort of 2184 cannabis-related emergency department encounters; from this, a subset was identified that comprised individuals presenting to the emergency department with cannabis use and a visit relating to suicide (including suicide attempt, suicidal ideation or suicidal self-harm). Initial search of the ‘chief complaint’ yielded 271 patients with a complaint coded as ‘depression/ suicidal’, and a search of ‘presenting complaint’ yielded another cohort of 271 patients coded as ‘depression/ suicidal’ and two patients with suicidal ideation. These identified patient groups were not entirely overlapping, and the medical record was hand searched for the reason for the emergency department visit. This search was included to capture any discharge diagnosis and other notes containing ‘suicide’ or variants of the word. Of these, two were noted to have ‘suicidal’ in the chief complaint description (one being ‘direct referral for consultation, suicidal’ and the other as ‘back pain, suicidal, overdose ingestion’), ten from ‘index diagnosis’, seven from ‘diagnosis description’, two from psychiatric consult diagnoses and one from the emergency department discharge summary. Consolidating these files and subsequent removal of duplicates resulted in 300 unique patients with both cannabis use and suicidality. From the index visit record, demographic data were collected, including sex, gender, age, ethnicity, employment status and living arrangements. Additionally, any concurrent diagnostic information was collected.

### Healthcare utilisation

Healthcare utilisation and further healthcare use pathway variables were collected for the index visit. These included the emergency department arrival method; whether the encounter resulted in a hospital admission, a discharge home, a referral or a transfer; and whether the patient left without being seen or left against medical advice. Healthcare usage for each patient was examined for the period of 24 months before the index encounter and 24 months following the index encounter, and consisted of the number of prior emergency department visits, whether the visit was cannabis related and the discharge diagnosis at each visit. To capture deaths, a chart review for death notes and obituary search through online means was conducted.

### Data analysis

Data were analysed through descriptive statistics to characterise the population, including demographic data, quantifying the most common complaints during visits other than the index visit, the outcomes of the index visit and outcomes of the pre- and post-index visits. Chi-squared analyses and Fisher exact tests were performed to further understand the relationship between index encounter time and outcome variables.

## Results

### Patients

The final sample consisted of any patients who reported cannabis use and had a presenting complaint related to suicide recorded in the Emergency Department Information System (*N* = 300, Table 1). This group was a subset of 2184 identified cannabis-related presentations from the two 6-month time periods. There were 1332 cannabis-associated emergency department encounters in the first time period and 852 in the second time period. Suicide attempts represented 13.7% of the cannabis-related emergency department presentations for any cause (medical or psychiatric). Patient ages ranged from 14 to 75 years (mean 30.05, s.d. = 11.837) There were nearly equivalent proportions of males (51.8%) and females (48.2%), with 51.7% of individuals self-identifying as men (Table 1). Most of the patients did have a family doctor at the time of the indexed encounter (70%). Housing was examined and most were securely housed (80.7%; *n* = 119). However, there was significant missingness in the housing data (not recorded in 181 charts). The main method of arrival was self, by a significant margin was self (34%), followed by roughly equivalent numbers arriving with a relative (23.7%) or by emergency health services (22.3%). These were the three main methods of arrival. See Table 1 for full demographic information.

**Table 1:** Demographics and emergency department arrival method for the complete cohort

	*n*	%
	*N* = 300	
Sex		
Male	156	51.8
Female	144	48.2
Gender		
Man	155	51.7
Woman	138	46
Transgender	7	2.3
Family doctor status		
Yes	211	70.3
No	84	28
Unknown	5	1.7
Housing situation		
Living with family	43	14.3
Living with partner	20	6.7
Living with roommate	17	5.7
Living alone	16	5.3
Insecurely housed	14	4.7
Unhoused	9	3
No data	181	60.3
Arrival method		
Self	102	34
Relative	71	23.7
Emergency health services	67	22.3
Police	33	11
Friend	25	8.3
Other	2	0.7

### Cohort comparisons

A series of analyses were performed to examine any differences between the two cohorts. To examine differences in categorical variables including gender, sex, site of indexed encounter and arrival method, a series of chi-squared tests were performed; these analyses did not show any significant group differences in demographic variables (Table 2). The first cohort (17 October 2018 to 30 April 2019) had 193 visits related to suicide, and the second (17 October 2020 to 30 April 2021) had 107 visits (Table 2). Independent samples *t*-tests were performed for continuous variables such as age, number of prior and subsequent encounters to the emergency department, the number of prior and subsequent encounters related to suicide alone, and the number of both prior and subsequent suicide-related encounters with concomitant reported cannabis use (Table 3). There was a significant difference (*t* = 2.23, *P* = 0.027) found for the number of emergency department encounters following the indexed visit relating to suicide between time frames, with patients who presented between 17 October 2018 and 30 April 2019 having significantly more visits (mean 1.04, s.d. = 3.17) compared with patients who presented between 17 October 2020 and 30 April 2021 (mean 0.49, s.d. = 1.05). There was also a significant difference in total number of return emergency department visits between the two cohorts (*t* = 2.05, *P* = 0.042).

**Table 2:** Chi-squared results of comparison between time periods

	17 October 2018 to 17 April 2019		17 October 2020 to 17 April 2021		*χ* ^2^ (d.f.)	*P*-value
	*n*	%	*n*	%		
Sex						
Male	98	51.78	58	54.21	0.324 (1)	0.57
Female	95	49.22	49	45.79		
Gender						
Man	96	49.74	59	55.14	4.136 (3)	0.23
Woman	90	46.63	48	44.86		
Transgender^ a ^	7	3.63	0	0		
Family doctor status						
Yes	140	72.54	71	66.36	2.144 (2)	0.34
No	51	26.42	33	30.84		
Unknown	2	1.04	3	2.80		
Housing situation						
Living with family	22	11.40	21	19.63	7.26 (6)^ b ^	0.30
Living with partner	13	6.74	7	6.54		
Living with roommate	10	5.18	7	6.54		
Living alone	10	5.18	6	5.61		
Insecurely housed	11	5.70	3	2.80		
Unhoused	8	4.15	1	0.93		
No data	119	61.66	62	57.94		
Site of indexed encounter						
Hospital A	153	79.27	73	68.22	7.17 (3)^ b ^	0.67
Hospital B	28	14.51	23	21.50		
Hospital C	12	6.22	9	8.41		
Hospital D	0	0	2	1.87		
Arrival method						
Self	59	30.57	43	40.19	7.10 (5)^ b ^	0.21
Relative	53	27.46	18	16.82		
Emergency health services	45	23.32	22	20.56		
Police	18	9.33	15	14.02		
Friend	17	8.81	8	7.48		
Other	1	0.52	1	0.93		

a. Transgender includes other gender identities including non-binary identifying individuals.

b. Analysis where some cells contain less than the minimum expected count of 5.

**Table 3:** Comparison of the two cohort time periods continuous variables analysis

	17 October 2018 to 17 April 2019 (*n* = 193)		17 October 2020 to 17 April 2021 (*n* = 107)		*t*-score (*P*-value)
	Mean	s.d.	Mean	s.d.	
Age (years)	30.89	11.76	28.52	11.87	1.67 (*P* = 0.97)
Number of prior encounters	3.53	7.31	2.92	4.70	0.78 (*P* = 0.44)
Prior encounters related to suicide	0.73	2.86	0.45	1.22	0.953 (*P* = 0.34)
Prior encounters related to suicide and cannabis	0.11	0.61	0.07	0.28	0.70 (*P* = 0.49)
Number of subsequent encounters	5.06	10.28	3.24	5.04	1.71 (*P* = 0.04)^*^
Number of subsequent encounters related to suicide	1.04	3.17	0.49	1.04	1.76 (*P* = 0.03)^*^
Number of subsequent encounters related to suicide and cannabis	0.14	0.54	0.08	0.39	0.94 (*P* = 0.34)

* Statistical significance at the 0.05 level.

Healthcare utilisation was examined for the total cohort to capture the rates of those who had prior and subsequent visits to the emergency department (see Supplementary Table 1 available at https://doi.org/10.1192/bjo.2025.10776). With respect to prior emergency department visits, 54.3% of the sample had presented to the emergency department in the 2 years before the indexed dates, with a mean number of prior visits of 3.3 (s.d. = 6.50) and a range of 0 (no prior visits) to 56 prior visits. Of the individuals with an emergency department visit before the index encounter, 29% of those presented once, 16.6% presented twice and 11.7% presented three times. Of those who presented to the emergency department at least once before the indexed encounter, 12.4% of prior encounters were related to suicide and 5.7% presented with a complaint relating to suicide and reported cannabis use in the emergency department records. In terms of subsequent visits, 74.3% of our sample had presented to the emergency department in the 2 years following the indexed encounter (mean 4.41, s.d.= 8.81, range 0–644), and of those who had subsequent visits, 16% presented once, 15.3% presented twice and 68.7% presented three or more times. Of those who presented following the indexed encounter, 31.6% of encounters were related to suicide, and 27.4% of those who presented with a complaint relating to suicide had a record of cannabis use in the emergency department chart (Supplementary Table 1).

An examination of factors possibly related to multiple presentations to the emergency department for a suicidal complaint was then conducted. A subset of the original cohort was created by looking for previous or subsequent presentations to the emergency department relative to the index encounter date that were recorded as concerns around suicide or suicidal ideation. Then, within this cohort, the medical record was searched for any record of cannabis use at that time. This resulted in two groups of patients who were subsequently compared: patients who had any prior or subsequent emergency department encounters where concerns related to suicide were expressly recorded in the medical record, even if that was not the presenting complaint (*n* = 117); and a second group of patients who had prior or subsequent encounters relating to suicide and were identified as a cannabis user in the same record (*n* = 34). To estimate this possible association with other patient characteristics, a series of chi-squared and Fisher exact tests were performed with sex, gender, family doctor status, living arrangement, site of indexed encounter, recorded diagnosis of a substance use disorder and method of arrival to the emergency department as outcomes. The presence of a record of at least one other emergency department visit (before or after the index visit) was significantly associated with the diagnosis of a substance use disorder (*χ* ^2^ (1) = 7.39, *P* < 0.05) when these two groups were compared. There were no other outcomes of statistical significance found between the aforementioned variables.

Further analyses were performed to focus on patient features potentially related to presentation. We examined a number of possible associations, such as the relationship between patients who had presented at any time relating to suicide or suicide and cannabis, and who left without being seen, left against medical advice, had a psychiatric consult as a result of the index encounter, had a previous mental health diagnosis (including overall mental health disorder, as well as specified developmental disorders, cognitive disorders, substance-related disorders, psychotic disorders, mood disorders, bipolar disorders, anxiety disorders, eating disorders, personality disorders, sleep disorders and other mental health disorders), who were on medication at the time of the encounter, had reported using alcohol during their index encounter or were insecurely housed at the time of the presentation. Of these characteristics, a significant relationship was found between patients who presented with a complaint relating to suicide and cannabis use at any time and diagnosis of a developmental disorder (*χ* ^2^ (1) = 11.21, *P* < 0.001), cognitive disorder (*χ* ^2^ (1) = 7.16, *P* = 0.007), substance use disorder (*χ* ^2^ (1) = 9.91, *P* = 0.002), psychotic disorder (*χ* ^2^ (1) = 8.65, *P* = 0.003), bipolar disorder (*χ* ^2^ (1) = 6.06, *P* = 0.014), personality disorder (*χ* ^2^ (1) = 21.25, *P* < 0.001), sleep disorder (*χ* ^2^ (1) = 9.46, *P* = 0.002) and diagnosis of another mental health disorder not classified (*χ* ^2^ (1) = 19.34, *P* < 0.001). Among patients who had any additional emergency department encounters related to suicide and who again reported cannabis use at that time, a significant relationship was only found with diagnosis of a personality disorder (*χ* ^2^ (1) = 11.73, *P* < 0.001) (Table 4).

**Table 4:** Analysis of any suicide attempt/ideation or suicide and cannabis presentation at any time in the pre- or post-index periods

	Any suicide attempt		*χ* ^2^ (d.f.)	*P*-value	Any suicide and cannabis		*χ* ^2^ (d.f.)	*P*-value
	Yes	No			Yes	No		
Sex								
Male	64	92	0.56 (1)	0.45	18	138	0.014 (1)	0.91
Female	53	91			16	1281		
Gender								
Man	65	90	1.73 (3)	0.631	18	137	0.92 (3)	0.82
Woman	50	88			16	122		
Transgender	2	4			0	6		
Other	0	1			0	1		
Family doctor status								
Yes	83	128	4.58 (2)	0.21	25	186	5.04 (1)	0.17
No	30	54			7	77		
Unknown	4	1			2	3		
Living arrangement								
Alone	6	11	10.83 (6)	0.094	3	14	4.87 (6)	0.56
With family	22	23			6	39		
With partner	7	12			2	17		
With roommate	4	14			2	16		
Unhoused	7	3			2	8		
Insecurely housed	7	5			3	9		
Unknown	64	115			16	136		
Site of indexed encounter								
Hospital A	87	139	5.99 (3)	0.11	28	198	1.53 (3)	0.68
Hospital B	23	28			5	46		
Hospital C	5	16			1	207		
Hospital D	2	0			0	21		
Arrival method								
Self	41	61	7.89 (5)	2.46	13	892	1.99 (5)	0.92
Relative	25	46			6	65		
Emergency health services	33	34			8	59		
Friend	7	18			2	23		
Police	14	19			5	28		
Other	1	1			0	2		
Left without being seen								
Yes	115	179	0.08 (1)	1.0 (Fisher exact)	1	5	0.17 (1)	0.52 (Fisher exact)
No	2	4			33	261		
Left against medical advice								
Yes	2	6	0.98 (1)	0.61	1	7	0.19 (1)	0.91
No	115	177			33	259		
Psychiatric consult at index								
Yes	55	73	1.48 (1)	0.22	18	110	1.66 (1)	0.20
No	62	110			16	156		
Previous mental health diagnosis								
Yes	103	155	0.66(1)	0.42	30	228	0.16 (1)	0.69
No	14	28			4	38		
Developmental disorder								
Yes	37	28	11.21 (1)	<0.001^**^	8	57	0.08 (1)	0.78
No	80	155			26	209		
Cognitive disorder								
Yes	16	9	7.17 (1)	0.007^*^	3	22	0.01 (1)	0.91
No	101	174			31	244		
Substance use disorder								
Yes	46	41	9.91 (1)	0.002^*^	13	74	1.59 (1)	0.21
No	71	142			21	192		
Psychotic disorder								
Yes	25	17	8.65 (1)	0.003^*^	5	37	0.02 (1)	0.80 (Fisher exact)
No	92	166			29	229		
Mood disorder								
Yes	77	121	0.003 (1)	0.96	24	174	0.36 (1)	0.55
No	62	40			10	92		
Bipolar disorder								
Yes	14	8	6.06 (1)	0.01^*^	3	19	0.13 (1)	0.73 (Fisher exact)
No	103	175			31	247		
Anxiety disorder								
Yes	53	72	1.04 (1)	0.31	16	109	0.46 (1)	0.50
No	64	111			18	157		
Eating disorder								
Yes	18	19	1.65 (1)	0.20	3	34	0.44 (1)	0.78 (Fisher exact)
No	99	164			31	232		
Personality disorder								
Yes	38	20	21.25 (1)	<0.001^**^	14	44	11.73 (1)	0.001^**^
No	79	163			20	222		
Sleep disorder								
Yes	21	12	9.46 (1)	0.002^*^	4	29	0.02 (1)	0.77 (Fisher exact)
No	96	171			30	237		
Other mental health disorder								
Yes	43	27	10.31 (1)	<0.001^**^	11	59	1.74 (1)	0.19
No	74	156			23	207		
Prescribed medication								
Yes	79	115	0.68 (1)	0. 40	24	170	0.59 (1)	0.44
No	38	68			10	96		

* Statistical significance at the 0.05 level.

** Statistical significance at the 0.01 or greater level.

We sought to identify completed suicides during the 2-year post-index period by two means: an obituary search was completed through both local and national obituary records, and patient charts were screened for death certificates. Within the full sample, only one death was recorded, and this was a completed suicide (*n* = 1, 0.33%).

## Discussion

This study sought to fill the gap in the literature surrounding the healthcare usage of patients who use cannabis and present to the emergency department with suicidal ideation and attempts. The only difference we observed between the two time periods was for the number of follow-up visits post-index, with the earlier cohort having more presentations to the emergency department from the period directly following legalisation than a similar time period 2 years later. This suggests that post-cannabis legalisation, there is not an ongoing increase in emergency department presentations for suicidal ideation and attempts. This is in line with other work in Canadian jurisdictions showing no increases in cannabis-related emergency department presentations overall post-legalisation.^17^ The second time period did overlap with the easing of COVID-19 restrictions in our jurisdiction, which had significantly affected overall emergency department volumes, with visit levels returning to pre-pandemic levels in 2022/2023 within Canada.^27^ The decrease in visit numbers for the second cohort is in line with the overall recorded 40% decrease in emergency department visit volume owing to COVID-19 restrictions between our two cohort time periods for our jurisdiction.^28^ However, as previously studied by Geda et al, when other factors were removed, cannabis use remained strongly associated with suicidal ideation during the COVID-19 pandemic.^20^ Combined, this suggests there is a stable base rate of presentations to the emergency department with suicidal ideation and attempts with documented cannabis use/history. Consequently, this remains a significant concern in the Canadian healthcare system.

Our finding of 13.7% of the overall cannabis-associated presentations being related to suicide is somewhat higher than studies looking exclusively at self-harm in the emergency department, which showed cannabis alone was involved in only 1.7% of self-harm cases, but our inclusion of depression/suicidal would encompass more presentations.^21^ Our finding is in line with studies using only psychiatric presentations as the denominator, which found suicidality encompassing greater than 20% of all acute psychiatric complaints following cannabis use.^13^ However, the potential role of cannabis use in suicide remains complex to study, given the lack of drug use information that is usually associated with completed suicides.

Although a significant portion of this study population presented to the emergency department on multiple occasions within a 2-year time frame before or after this index visit (54.3% in the 2 years before and 74.3% in the 2 years after), a smaller percentage of presentations were related to the same complaint. Of patients who had prior encounters, there were relatively few with similar complaints to the index visit (i.e. relating to either suicide or suicide with noted cannabis use) compared with the patients who had subsequent encounters. This may reflect a change in attitudes toward or documentation of cannabis use, with increased reporting and documentation as more time since legalisation has passed. It could also potentially be a marker of more careful record review noting before cannabis use for an individual with a documented suicidal ideation or attempt. Conversely, this may reflect a true change in the healthcare needs of patients with cannabis use, who may represent a population at increased risk of overall health problems requiring multiple emergency department presentations. We found relationships between clinical information and repeated encounters to the emergency department for suicide or suicidal ideation and reported cannabis use. Certain mental health diagnoses, including developmental disorder, cognitive disorder, substance use disorder, psychotic disorder, bipolar disorder, personality disorder and sleep disorder, were found to be related to multiple subsequent emergency department visits for suicide-related presentations, and thus individuals could be considered at greater risk when they present to the emergency department with these comorbid mental health and cannabis use profiles. Although clinicians working in the emergency department often pay particular attention to whether individuals have a concurrent mental health diagnosis at the time of a presentation relating to suicide, the added impact of having used cannabis may represent a potential point of intervention to attempt to mitigate the risks of recurrence. In this situation, patients could benefit from targeted interventions involving psychoeducation around the effects of cannabis and the potential interplay with suicidal ideation, to reduce the rates of subsequent presentations and decrease the strain on both patients and the emergency department. In bringing awareness to this relationship, practitioners may be better able to identify who is at an increased risk of recurrent suicide-related presentations; however, further research related to how to mitigate these is necessary. Our finding that most of the individuals in these cohorts had a history of prior emergency department visits likely related to cannabis use further shows the potential value of intervening. It is also of note that although there were numerous emergency department presentations for suicidal ideation and suicide attempt concurrent with cannabis use, we could only find one completed suicide in our cohort. This low rate of completed suicides is in line with previously published longitudinal research.^6^

### Limitations

Studying cannabis use or any recreational drug use and its association with suicide is challenging for a variety of reasons that we have outlined earlier. We chose one direction to further this research by examining suicidal ideation and cannabis use as it presents to the emergency department. However, many patients with suicidal ideation or attempted suicide may leave the emergency department without being seen or else against medical advice,^16^ all of which can result in skewed data through emergency department administrative databases. In addition, direct intoxication can confound the psychological assessment, given the nature of suicide. We also focused exclusively on creating cohorts based on cannabis use as a key characteristic because we were primarily interested in determining if these presentations would change over time, and also to see if there were prior encounters that would show the value for intervening around cannabis use in the emergency department. It is also worth acknowledging the significant impact the COVID-19 pandemic may have had on presentations within the second time frame from 17 October 2020 to 17 April 2021, as measures were still in place in Nova Scotia to curb the spread of COVID-19, albeit less strict than the initial lockdown. Some early research has suggested that within the first lockdown period from March to May 2020, emergency department presentations in Nova Scotia overall decreased by up to 40% compared with the previous year.^28^ Being that the timeline in this study is months following this first wave of COVID-19, this may help to explain why the second time frame had few subsequent presentations compared with the pre-COVID-19 time frame. However, it is overall unclear how this trend may have affected the number of presentations, so further investigations in this area are warranted.

In conclusion, individuals present to the emergency department with cannabis use less frequently than alcohol and, because of the often chronic nature of cannabis use in conjunction with patient presentation, cannabis use is not necessarily flagged as a concern. However, it is clear from our work that suicidal ideation is associated with cannabis in emergency department presentations, and further work remains to determine the frequency and causality links in acute versus regular chronic use.

## Supporting information

Simmons et al. supplementary material Simmons et al. supplementary material

## Data Availability

The data that support the findings of this study are available on request from the corresponding author, C.E.C. The data are not publicly available due to legislative restrictions related to privacy of research participants.

## Supplementary material

The supplementary material is available online at https://doi.org/10.1192/bjo.2025.10776
