# Supplementary material for: Understanding the role of cannabis in patients with suicidal ideation presenting to the emergency department: systematic chart review
Source: BJPsych Open. 2025 Sep 9;11(5):e199. doi: 10.1192/bjo.2025.10776 (PMC12451717; doi:10.1192/bjo.2025.10776)
Supplement: Simmons et al. supplementary material [file S205647242510776Xsup001.docx]

Suppl. Table 1. Summary of Prior and Subsequent ED Encounters

|  | Minimum | | | Maximum | Mean (SD) | |  |
| --- | --- | --- | --- | --- | --- | --- | --- |
| Prior ED Encounters | 0 | | | 56 | 3.31 (6.50) | |  |
| Prior ED Encounters with Cannabis Use explicitly noted | 0 | | | 5 | 0.32 (0.95) | |  |
| Prior ED Encounters Suicide | 0 | | | 28 | 0.63 (2.41) | |  |
| Prior ED Encounters with Cannabis Use and Suicide | 0 | | | 6 | 0.09 (0.52) | |  |
| Subsequent ED Encounters | 0 | | | 64 | 4.41 (8.81) | |  |
| Subsequent ED Encounters with Cannabis Use explicitly noted | 0 | | | 12 | 0.475 (0.07) | |  |
| Subsequent ED Encounters Suicide | 0 | | | 33 | 0.84 (1.52) | |  |
| Subsequent ED Encounters with Cannabis Use noted and Suicide | | 0 | 5 | | | 0.12 (0.28) | |
